# Control of Tungiasis through Intermittent Application of a Plant-Based Repellent: An Intervention Study in a Resource-Poor Community in Brazil

**DOI:** 10.1371/journal.pntd.0000879

**Published:** 2010-11-09

**Authors:** John Buckendahl, Jörg Heukelbach, Liana Ariza, Judith Dorothea Kehr, Martin Seidenschwang, Hermann Feldmeier

**Affiliations:** 1 Department of Microbiology and Hygiene, Campus Benjamin Franklin, Charité University Medicine, Berlin, Germany; 2 Department of Community Health, School of Medicine, Federal University of Ceará, Fortaleza, Brazil; 3 Post-Graduation Program in Medical Sciences, School of Medicine, Federal University of Ceará, Fortaleza, Brazil; Queensland Institute for Medical Research, Australia

## Abstract

**Background:**

Tungiasis, an ectoparasitosis caused by the female sand flea *Tunga penetrans*, is an important health problem in many impoverished communities in the tropics. Sand flea disease is associated with a broad spectrum of clinical pathology and severe sequels are frequent. Treatment options are limited.

**Methodology/Principal Findings:**

We assessed the effectiveness of the intermittent application of the plant-based repellent Zanzarin to reduce infestation intensity and tungiasis-associated morbidity in a resource-poor community in Brazil, characterized by a very high attack rate. The study population was randomized into three cohorts. Initially, during a period of four weeks, the repellent was applied twice daily to the feet of all cohort members. This reduced the number of embedded sandfleas to 0 in 98% of the participants. Thereafter members of cohort A applied the repellent every second week twice daily for one week, members of cohort B every fourth week for one week, and members of cohort C served as controls. Infestation intensity and tungiasis-associated morbidity were monitored during five months. The intermittent application of Zanzarin for one week every second week significantly reduced infestation intensity from a median 4 lesions (IQR 1–9) during the whole transmission season. In contrast, in cohort B (application of the repellent every fourth week) the infestation intensity remained twice as high (median 8 lesions, IQR 9–16; p = 0.0035), and in the control cohort C 3.5 times as high (median 14 lesions; IQR 7–26; p = 0.004 during the transmission season). Tungiasis-related acute pathology remained very low in cohort A (median severity score 2; IQR 1–4) as compared to cohort B (median severity score 5; IQR 3–7; p<0.001), and control cohort C (median severity score 6.5; IQR 4–8; p<0.001).

**Conclusions/Significance:**

Our study shows that in a setting with intense transmission, tungiasis-associated morbidity can be minimized through the intermittent application of a plant-based repellent.

## Author Summary

Tungiasis is a parasitic skin disease caused by the female sand flea *Tunga penetrans*. The disease is frequent in resource-poor communities in South America and sub-Saharan Africa and affects the poorest of the poor. Sand flea disease is associated with a considerable morbidity and may lead to tetanus in non-vaccinated individuals. The degree of morbidity depends on the intensity of infestation, i.e., the number of embedded sand fleas a person has. Since tungiasis is a zoonosis involving a host of animal reservoirs, and because an effective treatment is not at hand, in resource-poor settings elimination is not feasible. Preventing morbidity to develop is therefore the only means to protect exposed individuals from sand flea disease. Similar to other arthropods, sand fleas can be repelled before they penetrate into the skin. In this study we show that the intermittent application of a plant-based repellent, of which the major component is coconut oil, reduces the intensity of infestation dramatically during the whole transmission season and prevents tungiasis-associated morbidity from developing. The prevention can be performed at the household level by the affected individuals themselves with minimal input from the health sector.

## Introduction

Tungiasis is a common, but neglected health problem in economically disadvantaged communities in tropical and subtropical countries [1]–[5]. The female sand flea *Tunga penetrans* penetrates into the epidermis of its host, undergoes a peculiar hypertrophy, expels several hundred eggs for a period of three weeks, and eventually dies in situ [6]. In endemic areas constant reinfestation is the rule and affected individuals frequently harbor dozens, sometimes hundreds of embedded parasites [7]. Tungiasis is acquired peri-domiciliary, but also inside the house [8]. Different species of animals act as reservoirs [4].

The ectoparasitosis mainly affects marginalized populations in urban squatter settlements, in villages in the hinterland, and in traditional fishing communities along the littoral [1], [3], [8]–[13]. In these settings tungiasis is commonly associated with an important morbidity, such as intense inflammation of toes and heels, painful fissures, ulcers and abscesses, as well as deformation, and loss of nails [4]–[6], [13]–[16]. Difficulty in walking is common; gangrene and tetanus are life-threatening sequels [4], [14], [17]–[20].

Since it is virtually impossible to eliminate tungiasis as long as the precarious living conditions characteristic for impoverished communities exist, morbidity control remains the only option. There is no effective chemotherapy available to kill embedded sand fleas. Parasites need to be extracted surgically with a sterile instrument. However, this requires a skilled hand and good eyesight. In resource-poor communities surgical removal is inconsistently performed and causes more harm than good if not done correctly [6], [21], [22]. We have previously shown that a regular twice daily application of Zanzarin, a repellent based on coconut oil, for a period of three weeks, reduced the rate of newly embedded fleas by 92%, and reversed tungiasis-associated clinical pathology almost completely [20]. In this study we investigated, whether an intermittent application of the repellent protects inhabitants of an area with intense transmission against penetrating sand fleas during the transmission season, in Northeast Brazil a period of six month.

## Materials and Methods

### Study area

The study was conducted in five neighborhoods (Luxou, Morro das Sandra's, Placas, Morra da Vitória and Novo Rumo) of the shantytown Vincente Prinzón, a typical conglomeration of urban squatter settlements (favela) in Fortaleza, Northeast Brazil. The area is characterized by intense transmission of *T. penetrans* [7]. The five neighborhoods are located on dunes near to the Atlantic Ocean. The area had been occupied by landless poor (“sem terra”) in the beginning of the 1950’s [23]. The poor living conditions have been described previously [24]. In brief, most houses are constructed with recycled litter and do not posses a concrete floor. Illiteracy, unemployment, crime, alcoholism, drug traffic, abuse, as well as adolescent prostitution, and domestic violence are common. Sixty percent of the population have a monthly family income of less than two minimum wages (1 monthly minimum wage 300 Real ≈ 100 Euro) [23]. In 2005, about 20.000 people inhabited the shantytown and were served by a single primary health care center.

### Study design

A randomized controlled trial was carried out in the five neighborhoods between June and December 2005. This period (dry season) coincides with the high transmission season of *T. penetrans* resulting in an attack rate of up to 10 newly embedded sand fleas per persons per day in a similar setting nearby [25].

Individuals with tungiasis were identified with the assistance of community health workers. They were included in the study provided they had at least 5 embedded sand fleas in stage 1 to 4 of the Fortaleza Classification, or a similar number of sand flea lesions manipulated with a perforating instrument [26]. Individuals who intended to change their place of residence during the next six months were not eligible. Individuals with ulcerated lesions necessitating antibiotic treatment, and children less than one year were excluded. In total 142 participants were recruited.

The intensity of infestation and the degree of tungiasis-associated morbidity was assessed as described previously [16]. After the admission examination the participants were randomized into three cohorts (A, B and C). In the first phase of the study individuals received a twice-daily application of Zanzarin for a period of four weeks. It was anticipated that this reduced the number of embedded sand fleas to almost zero [20]. Thereafter participants were examined again and the degree of the tungiasis-associated morbidity was assessed. These data provided the baseline for the subsequent study phase.

During a period of five months members of Cohort A applied the repellent every second week twice daily for one week, and members of Cohort B every fourth week twice daily for one week. Cohort C served as control group and did not receive any protection.

During the intervention periods Zanzarin was applied by trained community health workers on the skin of the feet (up to the ankle) including the interdigital areas. The average volume applied was 3 ml per person and day (Calculation based on the number of 100 ml bottles used per day, divided by the number of treated individuals). Prophylaxis was performed in the morning between 6 and 8 a.m., and in the evening between 6 and 8 p.m. The exact time of application was recorded for each study participant. The application of the repellent was regularly checked by random visits to the households of the study participants by one of the investigators (J.B.). In addition, at each examination the participants were asked whether the repellent had been applied regularly. This ensured that the repellent was applied exactly as defined in the study protocol, not spilled, given away for money, or stolen. The participants were asked not to wash their feet for at least two hours after application of the repellent. However, they were allowed to take a shower whenever they wanted.

Unexpectedly, staff members were assaulted during the second part of the study. For safety reasons we decided to interrupt the regular follow-ups at calendar week 45. Monitoring was resumed at calendar week 47. Nonetheless, the application of the repellent was continued during this period.

### Zanzarin

The repellent used was Zanzarin, a lotion based on coconut oil (*Cocos nucifera*), jojoba oil *(Simmondsia chinesis)* and *Aloe vera* (Engelhard Arzneimittel GmbH & Co. KG, Niederdorfelden, Germany). The lotion is sold as a biocide with repellent activities against ticks and biting insects. The exact composition is described in an annexed file (Text S1). As Zanzarin has a peculiar odor, neither the investigator nor the patient were blinded to group assignment. Adverse reactions were interrogated and documented at each visit. A previous study has shown that Zanzarin, is highly effective in preventing the infestation with sand fleas [27].

### Clinical examination

Since tungiasis can occur at any area of the body [28], [29], the whole body surface was examined for the presence of immature, egg-producing or dead fleas, and manipulated lesions. Tungiasis lesions were classified according to the Fortaleza Classification [26]. The following findings were considered diagnostic for tungiasis:

– Flea in *statu penetrandi* (stage I)

– A dark and itching spot in the epidermis with a diameter of 1 to 2 mm, with or without local pain and itching (early lesion, stage II)

– Lesions presenting as a white halo with a diameter of 3 to 10 mm with a central black dot (mature egg producing flea, stage III)

– A brownish–black circular crust with or without surrounding necrosis of the epidermis (dead parasite, stage IV).

During monitoring the number of viable (stage I to III), and dead (stage IV) sand fleas, and the total number of sand flea lesions were determined. Clinical pathology was documented every four weeks. Lesions manipulated by the patient (such as partially or totally eliminated fleas leaving a characteristic crater-like sore in the skin), and suppurative lesions caused by the use of non-sterile perforating instruments, such as needles, and thorns, were documented as well. The exact topographic localization of each lesion, its stage, and appearance were documented on a visual record sheet.

Clinical pathology was assessed in a semi-quantitative manner using a previously elaborated severity score for acute tungiasis (SSAT), and a severity score for chronic tungiasis (SSCT) [16]. The SSAT score comprises the following signs and symptoms: erythema, edema, pain upon pressure or spontaneously, itching, sleep disturbance due to itching, difficulty walking as indicated by an altered gait; abscess, and suppuration as indicators of superinfection; fissures, and ulcers as characteristic chronic skin defects [12], [26]. The score can take a value from 0–24 points.

The SSCT ranges from 0 to 33 points and comprises the presence of nail deformation, nail loss, brilliant skin (an indicator of chronic edema), deformation of toes; hypertrophic nail rim, and perilesional desquamation; the latter two characteristics are indicators of repeated tungiasis experienced in the past [12], [16], [26].

### Randomization

Households were randomized using a permuted block design (block size six, allocation ratio 1∶1). An investigator not involved in the follow-up visits created the randomization code. A computer generated random list was used.

### Statistics

All data were entered into an Epi-Info database (CDC, Atlanta, Ver. 6.04d) and checked for errors, which might have occurred during data entry. The database was exported into SigmaStat and SigmaPlot (Systat Software GmbH, San José, Version 2007). The main outcome measure was the intensity of infestation, i.e. the number of sand flea lesions present at the time of examination. Secondary outcome measures were the severity score of acute, and the severity score of chronic pathology. As the variables assessed were not normally distributed and variances varied considerably, the median and the interquartile ranges were used to indicate the average and dispersion of data. To compare results between the cohorts, the Wilcoxon Signed Rank Test was used; for correlation analysis, Spearman’s Rho was calculated. In order to detect a difference of 50% in infestation intensity between cohorts A and C, a sample size of 42 individuals for each cohort was calculated (level of significance 95%, power of the test 80%). In order to compensate for drop-outs it was decided to recruit 140 individuals to the study.

### Ethical considerations

The study was approved by the Ethical Committee of the Federal University of Ceará, Brazil (43/05, SINESP) and was registered at Controlled-trials.com (ISRCTN16910507). Informed written consent was obtained from all participants and in the case of minors from the parents or legal guardians. At the end of the study, all participants as well as their household members were carefully examined for the presence of embedded sand fleas. Individuals with tungiasis were treated with Zanzarin twice daily for a period of three weeks, a measure effectively reducing the number of embedded sand fleas and the degree of clinical pathology to almost zero [20].

## Results

### Baseline

**Figure 1:**
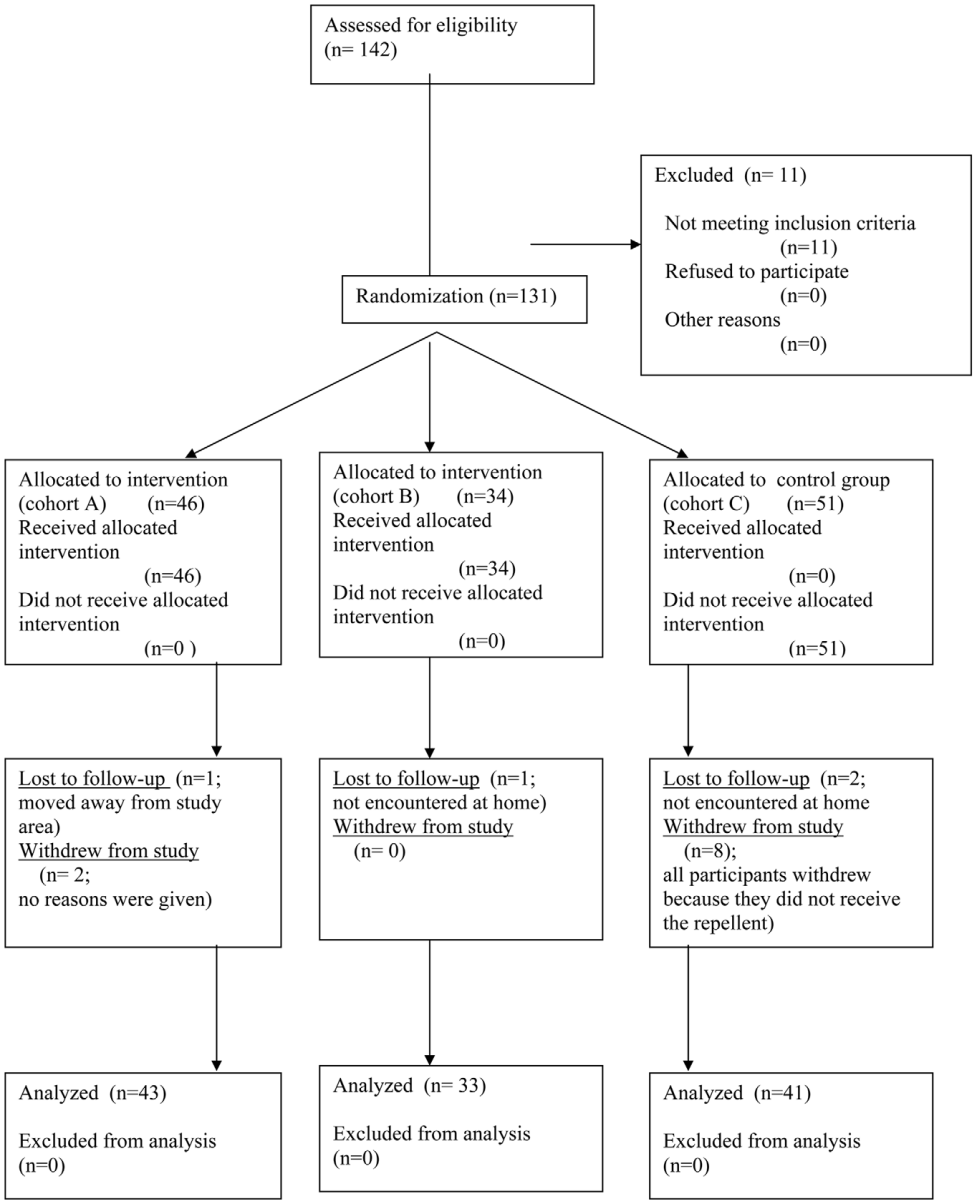
Flow diagram of the study.

The flow diagram of the study is depicted in Figure 1. The median age of all participants was 8 (range 1–66) with no difference between the cohorts. 43.9% of the participants were males, and 56.1% were females. The parasitological characteristics of the study population are depicted in Table 1. There was no difference between the three cohorts.

**Table 1:** Demographic and parasitological characteristics of the study population.

Variable	Cohort A(n = 45)	Cohort B(n = 34)	Cohort C(n = 43)
Age (median/range)	8 (4–12)	9 (5–16)	7 (5–10)
Sex ratio (females/males)	1.6	1	1.1
**Parasitological examination at admission**			
Total number of embedded sand fleas (median/IQR)	18 (9–31)	16 (12–34)	17 (10–25)
Viable lesions (median/IQR)	4 (2–14)	6 (3–12)	6 (4–10)
**Parasitological examination after initial intervention**			
Total number lesions (median/IQR)	0 (0–2)	0 (0–1)	0 (0–1)
Viable lesions (median/IQR)	0 (0–1)	0 (0–1)	0 (0–1)

### Initial intervention

After the initial intervention (application of Zanzarin twice daily in all cohort members during a period of four weeks) the infestation intensity decreased from a median of 17 (IQR 11–30), to a median of 0 (IQR: 0–1; p<0.001). Only two participants showed more than one embedded sand flea. This was paralleled by a drastic reduction of the SSAT: median before intervention 8.5 (IQR: 6–11), versus 0 (IQR: 0–1; p<0.001). The SSCT score was also reduced: median before intervention 12 (IQR: 9–15), versus 7 (IQR: 5–10; p<0.001). No adverse reactions to the repellent were reported.

### Intermittent intervention

#### Intensity of infestation

**Figure 2:**
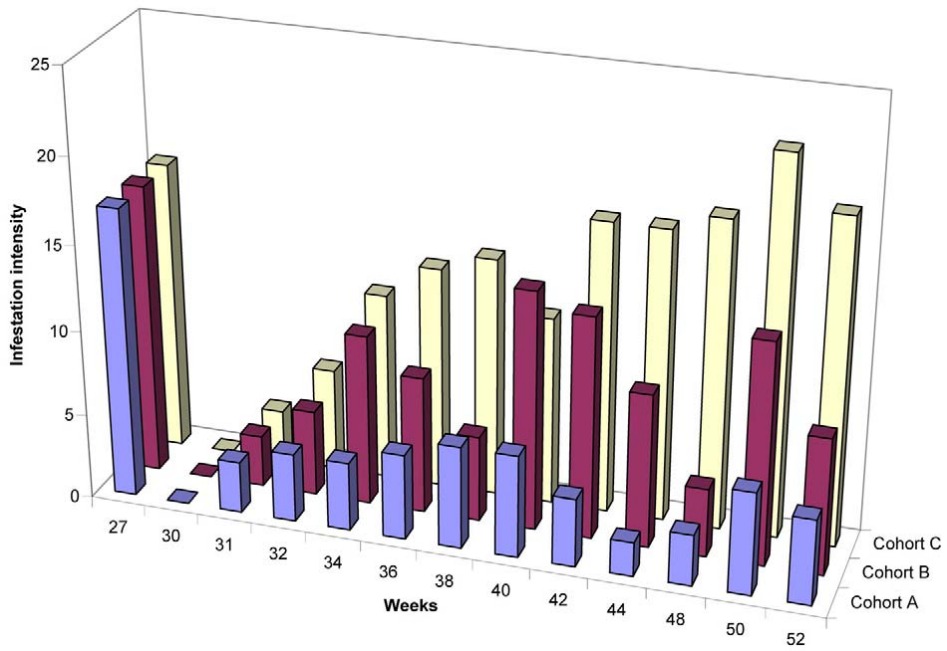
Intensity of infestation (Stage I-IV+ manipulated lesions) during the whole study period. The data indicate the number of median lesions per individual. No follow-ups were carried out between calendar weeks 45–47 (see Materials and Methods).

Figure 2 displays the total number of lesions (stage I-IV + manipulated lesions) during the whole study period. In cohort A (application of Zanzarin every second week for one week) the intensity of infestation rose to a maximum of 6 sand flea lesions per individual at calendar week 40 (IQR 2–8; p<0.001), thereafter decreased to 2 lesions (IQR 0–7; p<0.001) at calendar week 44. At the end of the study (calendar week 52), the intensity of infestation was 5 (IQR 1–10; p<0.001), as compared to calendar week 26. A similar pattern was observed in cohort B (application of Zanzarin every fourth week for one week), although the intensity of infestation remained much higher: a maximum of 9 lesions (IQR 3–18; p<0.002 (compared to cohort A)) was observed at calendar week 44, and 8 (IQR 4–16; p<0.001(compared to cohort A)) at calendar week 52.

In contrast, in the control cohort the intensity of infestation almost constantly increased during the transmission season, and reached a maximum of 19 sand flea lesions (IQR 10–34) at calendar week 50. Intensity of infestation between calendar week 31 and 52 was significantly higher in cohort C, as compared to cohort A (p = 0.004), and between cohort C and cohort B (p = 0.04). During this period intensity of infestation was also significantly higher in cohort B, as compared to cohort A (p = 0.03) (Figure 2).

#### Viable lesions

**Figure 3:**
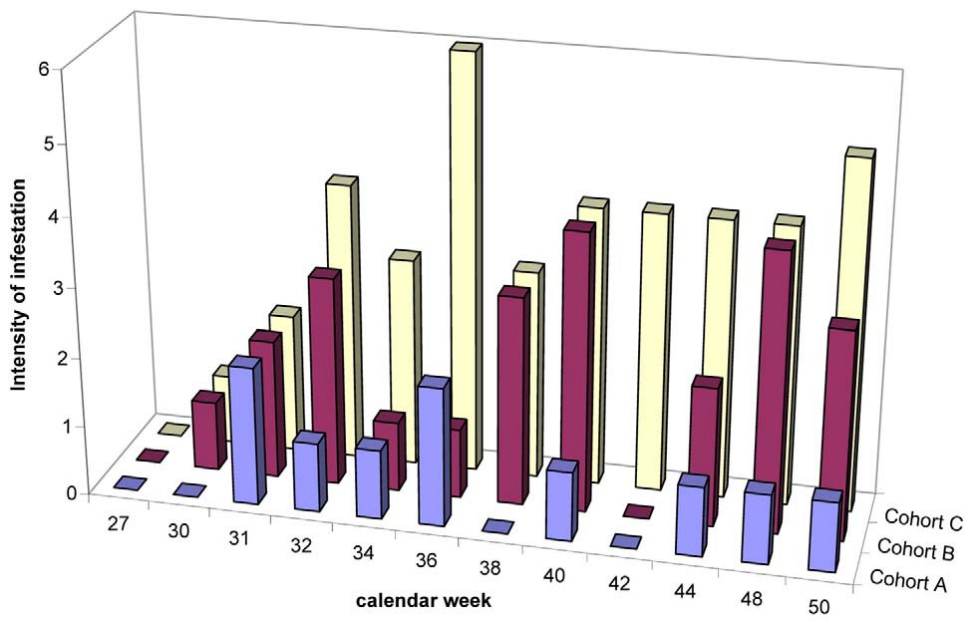
Number of viable lesions (stage I-III of the Fortaleza Classification) during the study period. No follow-ups were carried out between calendar weeks 45–47 (see Materials and Methods).

As seen in Figure 3 the number of viable lesions, indicating a recent penetration of sand fleas, remained very low during the intervention period (calendar week 31 to calendar week 52) in cohort A: median 1(IQR 0–3). In cohort B the median number of viable lesions was twice as high during this period: median 2 (IQR 1–5; p = 0.02). The highest number of viable lesions was observed in the control group: median 4 (IQR 1–8; p<0.001 (compared to cohort A); p = 0.002 (compared to cohort B)).

#### Manipulated lesions

**Figure 4:**
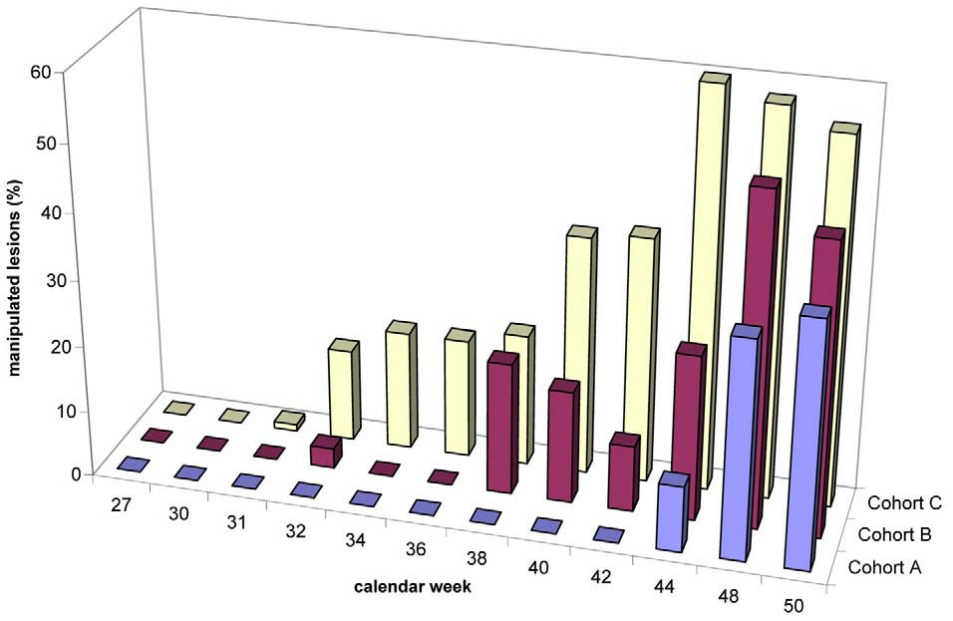
Proportion of manipulated lesions during study period. No observations were carried out between calendar weeks 45–47 (see Materials and Methods).

The proportion of manipulated lesions during the observation period is depicted in Figure 4. At baseline, 18% of all lesions had been manipulated by the patient or his caregiver, but the proportion dropped to zero after the first round of intervention. Whereas in cohort A the proportion of manipulated lesions remained zero until calendar 44, in the other cohorts the proportion of manipulated lesions started to rebound much earlier: cohort B in calendar week 40, control cohort C in calendar week 34. During the intervention period (calendar week 30 to 52) the proportion of manipulated lesions differed significantly between cohort A, and cohort C (p = 0.02), and between cohort B, and the cohort C (p = 0.045).

In cohort B and C the number of manipulated lesions correlated with the total number of lesions during this period: Cohort B rho = 0.67; p = 0.002, cohort C rho = 0.9, p<0.001. In cohort A no such correlation was observed.

#### Clinical pathology

**Figure 5:**
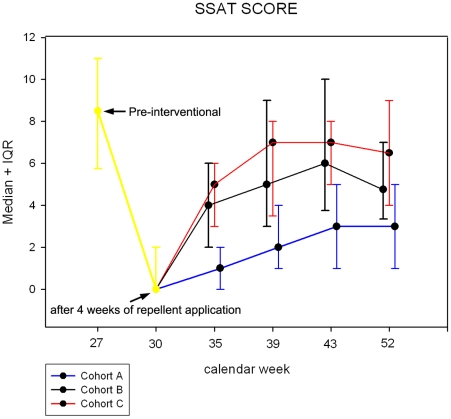
SSAT-scores (median and interquartile range) during the study period. During the first round of application of Zanzarin (twice daily for 4 weeks) the SSAT decreased in an identical manner in the three cohorts. For sake of clarity the data points of cohort B and C were shifted +/−0.1 calendar weeks on the x-axis, although the assessment of the SSAT was done simultaneously in the three cohorts.

The degree of clinical pathology was measured by the severity score for acute tungiasis (SSAT), and the severity score for chronic tungiasis (SSCT). Figure 5 shows the SSAT during the interval application of Zanzarin (calendar week 31 to week 52) in the three cohorts. In cohort A the median SSAT slightly increased from zero, the value reached after the initial four weeks of application of the repellent, to 2 (IQR 1 to 4) at the end of the study (p<0.001). This value favorably compared to a SSAT of 12 at admission to the study. In cohort B and cohort C the SSAT already started to increase sharply between calendar week 30, and calendar week 35 (p<0.001 and p<0.001), and was considerably higher, as compared to cohort A, at the end of the study (median 5 IQR 3–7; p<0.001 and median 6.5 IQR 4–8; p<0.001, respectively;).

**Figure 6:**
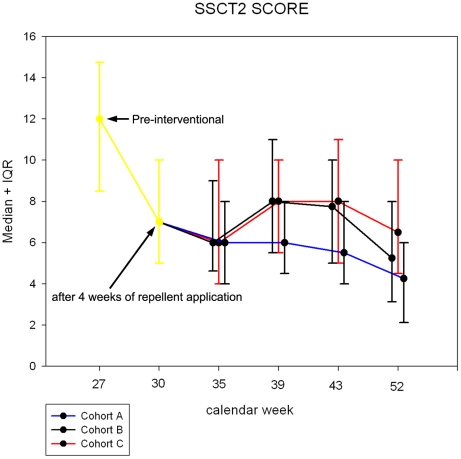
SSCT-Score during the study period. During the first round of application of Zanzarin (twice daily for 4 weeks) the SSCT decreased in an identical manner in the three cohorts. For sake of clarity the data points of cohort B and C were shifted +/−0.1 calendar weeks on the x-axis, although the assessment of the SSCT was done simultaneously in the three cohorts.

The pattern of the SSCT is shown in Figure 6. In cohort A the degree of chronic pathology significantly declined from a baseline value of 12 (IQR 9–15), to 4 (IQR 2–6; p<0.001) at the end of the study. In cohort B the decrease of the SSCT was similar: 12 (IQR 9–15) at baseline, to 5 at calendar week 52 (IQR 3–8;p<0.001). The lowest reduction of the SSCT was noted in Cohort C: 12 (IQR 9–15) at baseline, to 6.5 (IQR 5–10; p<0.001) at calendar week 52.

## Discussion

In this study we investigated, whether the intermittent application of Zanzarin keeps the infestation rate at an acceptable low level, and prevents severe clinical pathology to develop during the transmission season, which coincides with the dry season of the year in most endemic areas [15], [26]. In Northeast Brazil transmission occurs mainly between July and December, i.e. a period of 6 months.

Since infestation rate and infestation intensity are closely related [8], [16], and assessment of the infestation rate is very laborious, we determined the infestation intensity in three cohorts at regular intervals, together with the degree of tungiasis-associated pathology. In cohort A (application of Zanzarin every second week for one week) the number of embedded sand fleas only slightly increased during the transmission season. In cohort B (application of the repellent every fourth week for one week) the intensity of infestation was considerably higher during this period. However, even the long intervention-free intervals resulted, at the end of the transmission season, in an infestation intensity of approximately half the number of embedded sand fleas found in the control cohort (Figure 2).

If sand fleas are effectively repelled, only a few new parasites will penetrate per unit of time. This is reflected by a low number of viable lesions (stage I to III of the Fortaleza classification). In fact, the median number of viable sand flea lesions per individual showed different patterns in each of the cohorts. Whereas in cohort A the median number of viable sand fleas was 1, in cohort B – and even more so in cohort C - the number of viable sand flea lesions increased in a step-wise manner during the intermittent application of the repellent (Figure 3). These findings indicate that after the application of Zanzarin for one week a residual effect of the repellent seems to occur for a couple of days, but that an interval of four weeks without the application of the repellent is too long to prevent sand fleas invading the skin. Previous observations had raised the expectation that a residual effect may persist for more than one week [20].

The irritation and pain caused by embedded sand fleas is the reason why affected individuals try to get rid of the parasites with sharp instruments. Supposedly, the more sand fleas penetrate and embed per unit of time, the higher is the proportion of lesions manipulated with instruments. Hence, an effective repellent will be reflected by a low percentage of manipulated lesions. Indeed, the effectiveness of Zanzarin to prevent a high intensity of infestation to build up, is mirrored by the pattern of manipulated lesions in the three cohorts. In cohort A, manipulated lesions were completely absent until almost the end of the transmission season, whereas in cohort C the number of manipulated lesions started to increase almost constantly after the end of the initial intervention, and with a certain delay also in cohort B (Figure 4).

As expected, the almost complete interruption of transmission in cohort A prevented severe pathology to develop. This is mirrored by a decrease between 64% and 88% of the SSAT score during the transmission season, as compared to the degree of acute pathology at admission (Figure 5). A median SSAT score of 2 points at the end of the study in this cohort indicates an insignificant degree of acute pathology [20]. When the intervals between the application of Zanzarin were extended to three weeks, the degree of clinical pathology developing during the transmission season almost doubled, as compared to cohort A, and was only slightly less than in the control group (Figure 5). Hence, regular application of Zanzarin every second week is necessary to reduce clinical pathology to an insignificant level.

The interval application of Zanzarin was less effective in reducing the presence of chronic pathology (Figure 6). This is plausible, since chronic pathology, such as nail deformation and fibrosis of the skin around the nail rim, needs long time without new infestations, to resolve at least partially [20]. However, it is important to note that the application of the repellent every second week significantly reduced the SSCT score during the follow-up period, as compared to the value at admission.

Based on an average of 3 ml of Zanzarin applied per individual per day, a member of Cohort A needed a total of 210 ml of the repellent for the whole transmission season. If Zanzarin is bought in bulk quantity, 10–12 US$ would be sufficient to protect one person against the debilitating sequels of tungiasis during the whole transmission season. Since children and the elderly are particularly affected by severe manifestations of sand flea disease [12], prophylaxis could be targeted to these population groups, reducing the costs of the repellent. The application of a repellent is a simple and sustainable means to relief the poorest of the poor from a scourge that reappears each year with the beginning of the dry season. Prevention could be achieved by preparing the repellent from local coconuts by the affected individuals themselves with minimal input from the health sector. Taken together, the intermittent application of Zanzarin, twice daily for one week every second week, effectively interrupts transmission of *T. penetrans* in an area with a high attack rate, and prevents severe morbidity to develop.

## Supporting Information

Checklist S1 CONSORT checklist.
(0.19 MB DOC)

Protocol S1 Clinical trial protocol.
(0.19 MB DOC)

Text S1 Compounds of Zanzarin.
(0.02 MB DOC)
