# Supplementary material for: Control of Tungiasis through Intermittent Application of a Plant-Based Repellent: An Intervention Study in a Resource-Poor Community in Brazil
Source: PLoS Negl Trop Dis. 2010 Nov 9;4(11):e879. doi: 10.1371/journal.pntd.0000879 (PMC2976681; doi:10.1371/journal.pntd.0000879)
Supplement: Protocol S1 — Clinical trial protocol. (0.19 MB DOC) [file pntd.0000879.s002.doc]

**Departamento de Saúde Comunitária**

**Faculdade de Medicina**

**Universidade Federal do Ceará**

**Fundação de Educação e Saúde Mandacaru**

**Aplicação intermitente de um repelente natural para reduzir a intensidade de infestação com *Tunga penetrans* e severidade de tungíase (*bicho de pé*) em uma área endêmica**

**PROJETO DE PESQUISA**

**Janeiro 2005**

### Sinopse

A tungíase (*bicho de pé*), causada pela penetração da pulga *Tunga penetrans* na epiderme do hospedeiro, é freqüentemente associada com morbidade severa em comunidades endêmicas. Infecções secundárias, perda de unhas e dificuldade em andar e pegar são comuns.

Um estudo recente mostrou que a aplicação de um repelente natural a base de óleo de coco nos pés duas vezes ao dia reduziu a taxa de infestação (número de pulgas novas penetradas por dia) em quase 90%. Simultaneamente, a morbidade causada por tungíase foi reduzida a níveis insignificantes.

O estudo presente avaliará o impacto da aplicação intermitente profilática de um repelente natural durante todo o período de transmissão da *T. penetrans*. Três diferentes esquemas serão comparados a respeito do seu efeito na infestação, proporção de lesões vitais e grau de patologia associada.

Os resultados ajudarão no planejamento de medidas custo-efetivas de controle de tungíase e abrirão novos caminhos para aplicação do repelente em comunidades afetadas.

**Objetivos**

Objetivo geral

- Comparar a efetividade de diferentes esquemas de aplicação profilática de um repelente natural a base de óleo de coco na redução da intensidade de infestação e morbidade em uma área de alta transmissão.

Objetivos específicos

- Reduzir a patologia causada pela tungíase na área de estudo
- Identificar a freqüência mínima do uso do repelente para reduzir morbidade associada a tungíase
- Abrir novos caminhos para a realização de medidas de controle custo-efetivas para o controle de tungíase

**Introdução**

Conhecimento atual sobre a tungíase

A tungíase é causada pela penetração da pulga fêmea *Tunga penetrans* na epiderme do homem e de diversos animais*.* Em 1525, a tungíase foi inicialmente descrita no Haiti onde conquistadores espanhóis eram freqüentemente acometidos pela doença (1;2). A primeira descrição científica da *T. penetrans* é da autoria de Aleixo de Abreu, um médico português a serviço do governador no Brasil no início do século XVII (3).

Originalmente a pulga era encontrada somente na América Latina e no Caribe. Acredita-se que o ectoparasita tenha sido inadvertidamente introduzido na África tropical em 1872 por uma carga de cascalho contaminada transportada pelo navio *Thomas Mitchell* do Brasil para Angola (2;4-6). A partir daí, a *T. penetrans* disseminou-se rapidamente ao longo da costa oeste africana e então para toda a região subsaariana (4-7).

Atualmente a pulga está distribuída em toda a América Latina, Caribe e África subsaariana. Na América Latina é encontrada do México ao norte da Argentina e Chile (8). O Brasil está particularmente afetado: a tungíase ocorre do Amazonas ao Rio Grande do Sul (8-12).

As pulgas adultas vivem livremente no ambiente, sendo que a pulga fêmea penetra na epiderme do seu hospedeiro: homem, gato, porco e muitos outros animais (8;13). Dentro de 24 horas após a penetração na epiderme, percebe-se uma reação inflamatória local e dois a três dias depois aparece a dor. A pulga necessita de 8 a 12 dias para atingir a maturidade. Durante esse processo, o seu abdome aumenta de tamanho consideravelmente e eventualmente atinge um diâmetro de um cm contendo cerca de 200 ovos. As pulgas podem viver no seu hospedeiro e expelir ovos por várias semanas. Após todos os ovos serem expelidos, a pulga morre, resseca-se *in situ* e eventualmente seus restos são expelidos. Uma pequena cicatriz permanece limitada à epiderme, que desaparece com o tempo (14).

Sem tratamento apropriado, são comuns as infecções secundárias (15). Em áreas de baixa cobertura vacinal, o tétano é uma complicação comum em crianças (16-19). Sepse, linfedema, gangrena, perda das unhas e auto-amputação de dígitos já foram descritos (8). Infestação severa com centenas de pulgas podem causar lesões *honeycomb-like* (20;21).

Nas áreas endêmicas, o diagnóstico da tungíase é normalmente realizado por inspeção macroscópica da lesão. Um dia após a penetração, percebe-se um pequeno ponto vermelho. Nos dias seguintes, a fêmea grávida é percebida na epiderme como uma mancha branca com um ponto negro central, o que representa os segmentos posteriores abdominais. Após a morte da pulga, as lesões são cobertas com uma crosta preta consistindo presumivelmente de sangue coagulado e debris.

O controle da doença se torna bastante complexo por causa da existência dos reservatórios animais domésticos (cão, gato) e selvagem (rato) (13;22).

Sem dúvida, a tungíase é ainda uma doença disseminada de grande relevância individual e em saúde pública no nordeste do Brasil. A doença tem sido negligenciada por todos: população, profissionais e autoridades de saúde. É imperativo aumentar o conhecimento científico sobre o tratamento da *T. penetrans* para se evitar as seqüelas debilitantes que o ectoparasita pode causar.

### Terapia e profilaxia da tungíase

Terapia convencional consiste em remover a pulga penetrada com uma agulha estéril e aplicação tópica de um antibiótico, se houver infecção secundária (8). Se a pulga rompe na tentativa de removê-la com uma agulha, espinho ou outro objeto pontiagudo não estéril, poderá ocorrer inflamação severa.

Vários autores descreveram terapias empíricas com drogas para a tunguíase, mas como a maioria desses estudos não foi controlado adequadamente, não há conclusão sobre suas eficácias (23-26). Em um estudo controlado em Fortaleza, o uso tópico de ivermectina, tiabendazol, metrifonate e óleo de mamona mostraram somente um efeito mínimo em pacientes severamente infestados (27). O uso oral de ivermectina também não se mostrou eficaz no tratamento da tungíase (28). Como o tratamento da tungíase parece não ser uma estratégia efetiva para reduzir a morbidade associada em comunidades endêmicas, medidas profiláticas, como o uso de um repelente, pode ser uma medida de controle mais promissora.

Tradicionalmente, habitantes de áreas endêmicas têm usado vários tipos de remédios tradicionais como repelente. Em Fortaleza, são utilizados uma mistura de cera com querosene, óleos etéricos e extratos de várias plantas. Por exemplo, o óleo de rícino, chamado “mamona’ pelos habitantes, é misturado com óleo de coco e aplicado freqüentemente nos pés.

Uso de repelente natural

Após os resultados promissores de um estudo piloto realizado com um repelente natural a base de óleo de coco por esse grupo de pesquisa em quatro indivíduos (29), foi realizado um estudo com desenho *cross-over*. O estudo mostrou que uma aplicação duas vezes ao dia reduziu a taxa de ataque de *T. penetrans* em cerca de 90% em uma área de alta transmissão (artigo para publicação submetido). A redução da taxa de ataque aconteceu paralelamente com uma redução da severidade da doença. O efeito profilático continuou por um certo tempo após a descontinuação da aplicação. Posteriormente, a taxa de ataque e severidade da doença aumentaram lentamente. Entretanto, esses dois parâmetros permaneceram abaixo do nível de base durante pelo menos cinco semanas após descontinuação da aplicação, apesar da transmissão na área de estudo continuar alta.

O repelente usado é uma loção biológica à base de coco, aloe vera (babosa), jojoba, pantenol (derivado de vitamina B) e vitamina E (vide material e métodos).

No estudo citado, não foram relatados efeitos colaterais consideráveis.

**Justificativa**

A tungíase mostra uma variação sazonal com quase nenhuma transmissão durante a época de chuva (30). Durante a época seca, a taxa de ataque aumenta, e o número de pulgas penetradas acumula, sendo que na estação chuvosa não acontecem novas lesões. Conseqüentemente, a intensidade da infestação diminui durante esse período.

No outro lado, é conhecido que a severidade da doença (dor, edema, supuração, úlcera, deformação de unhas etc.) é diretamente relacionada ao número de parasitas penetrados na pele.

Como em comunidades endêmicas típicas a tungíase não pode ser erradicada a curto prazo, a redução da morbidade durante o período de transmissão para um nível aceitável traria um importante benefício para a população afetada.

Esse estudo visa comparar diferentes esquemas de aplicação profilática de um repelente natural em relação à efetividade em reduzir intensidade de infestação e, subseqüentemente, de morbidade durante o período de transmissão.

No final do estudo, além de ter reduzido a morbidade na área de estudo, será conhecida a freqüência mínima eficaz de aplicação do repelente para reduzir a patologia associada a tungíase a um nível insignificante em uma área de alta transmissão. Isso permitirá a realização de controle custo-efetivo de tungíase em comunidades carentes e abrirá caminhos novos para o controle da doença em larga escala.

# Área e população de estudo

Pretende-se realizar o estudo na favela Serviluz/Vicente Pinzón II, uma favela típica em Fortaleza, capital do Estado do Ceará. A favela é localizada à beira-mar, onde migrantes pobres do interior do Estado começaram a habitar as dunas no início dos anos 50. A área tem uma população total de 20.000 pessoas. 97% das famílias tem energia elétrica, 70% tem acesso a água encanada e 60% tem uma renda familiar de menos de dois salários mínimos. A taxa de analfabetismo em adultos é de 30%.

Na área de estudo as doenças de pele são hiperendêmicas: a tungíase ocorre em 34% da população, sendo a faixa etária de 5 a 9 anos a mais atingida com 58% de prevalência (10). O contato do grupo de pesquisa com os líderes comunitários da área é bem estabelecido. A comunidade já cooperou em outros estudos sobre doenças parasitárias de pele.

A sede da Fundação Mandacaru está situada no centro da área de estudo.

Tamanho de amostra

Para detectar uma diferença de infestação de 50% entre coorte 1 e coorte 2, um tamanho de amostra de 52 indivíduos será necessário em cada grupo (nível de significância de 5%, poder de 80%). Assumindo uma taxa de perda de seguimento de 20% durante seis meses, 65 a 70 indivíduos serão incluídos em cada coorte, o que resultará em número total de 195 a 210 indivíduos para os três coortes.

### Desenho do estudo

O estudo é desenhado como um ensaio de intervenção com três braços. Três coortes serão formadas e seguidas durante o período de transmissão inteira, i.e. para um período de seis meses. Cada coorte consiste em 65 a 70 indivíduos de uma micro-área descrita da comunidade.

Durante o período de três semanas, serão realizados os exames de base. Posteriormente, o repelente natural será aplicado duas vezes ao dia durante um período de três semanas nos três coortes. Isso reduzirá a intensidade de infestação e o grau de patologia clínica para um nível insignificante. Ao final da primeira fase, se ainda houver pulgas penetradas, estas serão retiradas manualmente sob condições estéreis.

Após a primeira fase de intervenção, os três coortes serão randomizados para diferentes esquemas de intervenção:

- coorte 1: nenhuma intervenção adicional.
- coorte 2: após um intervalo de uma semana sem repelente, será realizada uma aplicação do repelente uma vez ao dia durante uma semana. Essa medida será repetida a cada segunda semana até o final do estudo (significa em total 10 semanas de aplicação).
- coorte 3: após de um intervalo sem repelente de três semanas, o repelente será aplicado uma vez ao dia durante uma semana seguida por um intervalo sem repelente de três semanas. Essa medida será repetida até o final do estudo (significa em total 5 semanas de aplicação).

Simultaneamente, a transmissão nas três micro-áreas será avaliada através de ratos-sentinela que serão colocados em gaiolas na área (ratos Wistar de laboratório).

Ingredientes do repelente natural

O repelente será feito em farmácia de manipulação à base das seguintes substâncias com concentração em ordem decrescente:

**1. Ácido capróico**

Descrição: Ácido graxo; obtido do óleo de coco

Função: Emulsificante

**2. Óleo de coco refinado**

Descrição: Obtido do coco seco (*Cocos nucifera*)

Função: Repelente

**3. Ésteres de Jojoba**

Cera de Jojoba, líquida

Descrição: Obtida por expressão de sementes maduras de *Simmondsia chinensis* (Buxeraceae).

Função: substância protetora de pele

**4. Acetato de α tocoferol**

Descrição: Acetato de vitamina E.

Função: Antioxidante

**5. Água**

Descrição: água destilada

Função: Veículo

**6. Ceteareth-12**

Macrogolcetylstearylether (12), Eumulgin B1

Função: Emulsificante

**7. Ceteareth-20**

Macrogolcetylstearylether (20-22), Eumulgin B2

Função: Emulsificante

**8. Metilparabeno**

4 metil hidroxibenzoato

Função: Conservante

**9. Propilparabeno**

Propyl-4-Hydroxybenzoato

Função: Conservante

**10. Estearato de potássio**

Função: Emulsificante

**11. Aloe Barbadensis**

Gel de Aloe vera 10:1, descolorido

Descrição: Obtido por expressão das folhas de *Aloe barbadensis* (Liliaceae).

Função: substância protetora de pele (hidratante e antiinflamatório)

**12. Tromethamine**

Trometamol, Tris(hydroxymethyl)aminomethan

Descrição: Álcool de aminas

Função: meio neutro básico, tampão

**13. Pantenol**

Dexapantenol

Descrição: Pró-vitamina B

Função: substância protetora de pele. O dexapantenol estimula a reepitelização e é antiinflamatório.

**14. Perfume**

Óleo de perfume BLUE WATER-Y (Nr. 0/279271)

Descrição: mistura complexa de diferentes álcoois, aldeídos, ésteres etc.

Função: corretivo de odor

# Exame de pacientes e medidas de desfecho

Antes, durante e após as medidas profiláticas serão documentados o estágio, a localização, o número de lesões e a presença de complicações. Os pacientes serão interrogados sobre sintomas como odor e prurido com o uso do repelente. Exames de seguimento serão realizados semanalmente durante o período de transmissão, i.e. cerca de seis meses. Será realizada foto-documentação antes e após o tratamento. Vale salientar que a privacidade do paciente será rigorosamente observada.

Caso sejam observadas outras doenças infecciosas ou não infecciosas que necessitem de tratamento, os pacientes serão encaminhados para o Posto de Saúde Aída Santos e Silva*.*

O efeito da aplicação intermitente será avaliado através dos seguintes parâmetros e comparado entre os grupos:

- prevalência longitudinal da tungíase (dias sem lesões vitais) no exame de base em comparação com a prevalência longitundinal durante a intervenção
- intensidade de infestação no exame de base em comparação com a intensidade de infestação no seguimento
- proporção de lesões vitais (Estágio II e III segundo *Fortaleza Classification*) no exame de base em comparação com a proporção de lesões vitais durante o seguimento
- grau de patologia clínica no exame de base em comparação com patologia clínica durante o seguimento

Para excluir que a variação desses parâmetros seja causada pela mudança temporal da taxa de ataque, ratos sentinela serão examinados durante todo o período de estudo.

As observações dos membros das famílias sobre a redução da taxa de ataque de pulgas de animais e de pernilongos também serão documentadas.

# Critérios de inclusão e exclusão

Critérios de inclusão

Serão incluídos pacientes da área de estudo, de ambos os sexos e todas as faixas etárias que assinem o consenso escrito pós-informação e que tenham pelo menos dez pulgas penetradas no exame de base.

Os indivíduos serão recrutados com a ajuda dos líderes comunitários e agentes de saúde.

Critérios de exclusão

Serão excluídos pacientes com menos de 10 lesões no exame de base ou que não assinem o consenso escrito livre. Pacientes com superinfecção severa serão excluídos do estudo e tratados adequadamente.

# Documentação de efeitos adversos e aceitabilidade

Sintomas que indiquem efeitos adversos relacionados ao tratamento serão interrogados a cada exame. Os efeitos adversos serão documentados através de formulário padronizado. Adicionalmente, os pacientes serão interrogados sobre a aceitabilidade do repelente como agente profilático.

Pacientes apresentando eventos adversos receberão assistência profissional e, caso necessário, terapia adequada.

Pacientes que apresentem efeitos colaterais em que seja necessária a suspensão do tratamento atual ou a instituição de terapia adequada medicamentosa não continuarão no estudo, sendo continuada a assistência médica. Todos os pacientes com infecção secundária como conseqüência da tunguíase receberão tratamento com antibiótico tópico (pomada de neomicina/bacitracina) ou oral (ampicilina). A assistência médica continuará mesmo que o paciente seja excluído do estudo.

**Análise de dados**

Todos os dados serão computadorizados usando o programa Epi-Info, versão 6.04d. A análise dos dados será realizada pelos integrantes do grupo de estudo.

### Considerações éticas

Toda pesquisa deve ser conduzida com três princípios éticos básicos: respeito pela pessoa, benefício para o paciente e justiça.

O respeito pela pessoa trata principalmente do conceito da autonomia, e afirma-se aqui que em nenhuma etapa será ferida a sua capacidade de deliberarem sobre suas escolhas pessoais, já que as informações serão mantidas em sigilo e trabalhadas de forma conjunta e aleatória. O benefício para o paciente refere-se à obrigação ética de maximizar benefícios e minimizar danos e prejuízos. Já firmada a relevância da pesquisa para o Estado, observa-se que inexistem danos ou prejuízos a qualquer das partes envolvidas. Há ainda a justiça, que trata da obrigação ética de tratar cada pessoa com o que é moralmente certo e adequado. Nos estudos propostos, desconhece-se onde este preceito esteja quebrado.

Todos os dados colhidos serão manuseados com a maior confiança. Os nomes dos pacientes não aparecerão em publicações nem serão acessíveis a terceiros. Os dados somente serão utilizados para fins dessa pesquisa.

O consentimento pós-informação escrito será obtido de todos os participantes do estudo ou de seus responsáveis utilizando-se a ficha do anexo. Qualquer participante poderá sair do estudo a qualquer momento sem nenhuma desvantagem.

Após a finalização do estudo, todas as famílias participantes receberão uma cesta básica.

**Risco/benefício**

Como a substância será aplicada de forma tópica, é de origem natural e está sendo utilizada em milhares de pessoas como repelente contra insetos, o risco do estudo proposto pode ser considerado como muito baixo. Vale ressaltar que a aplicação do repelente não é uma medida terapêutica, mas profilática.

Em estudo recentemente realizado, a aplicação do repelente se mostrou altamente seguro sem efeitos colaterais consideráveis. Além disso, naquele estudo a aplicação foi realizada duas vezes ao dia em contraste com a aplicação única diária no estudo presente.

O benefício para os participantes do estudo é a redução da patologia associada a tungíase e a redução da taxa de ataque na comunidade. Os resultados trarão informações de grande valor para a Saúde Pública no Estado. Será conhecido, por exemplo, qual a freqüência mínima de aplicação eficaz para o repelente reduzir patologia associada a tungíase de forma significativa. Esse conhecimento permitirá a futura realização de medidas de controle custo-efetivas para a tungíase em comunidade carentes. Como o repelente é à base de coco, aloe vera (babosa), jojoba, pantenol (derivado de vitamina B) e vitamina E, substâncias facilmente encontrada em nível local, este poderá ser fabricado localmente.

**Publicação de resultados**

Os resultados da pesquisa - favoráveis ou não favoráveis - serão de conhecimento público, sendo apresentados em congressos nacionais e internacionais, além de publicados em jornais indexados nacionais e internacionais.

**Cronograma/duração da pesquisa**

O trabalho de campo está planejado para iniciar em Maio/Junho de 2005 (início da alta transmissão da tungíase) e para terminar em dezembro de 2005 (duração total de 28 semanas).

As fases diferentes do estudo estão detalhadas na tabela:

| **Semana** | | | | | | | | | | | | | | | | | | | | | | | | | | | | |
| --- | --- | --- | --- | --- | --- | --- | --- | --- | --- | --- | --- | --- | --- | --- | --- | --- | --- | --- | --- | --- | --- | --- | --- | --- | --- | --- | --- | --- |
| coorte | 1 | 2 | 3 | 4 | 5 | 6 | 7 | 8 | 9 | 10 | 11 | 12 | 13 | 14 | 15 | 16 | 17 | 18 | 19 | 20 | 21 | 22 | 23 | 24 | 25 | 26 | 27 | 28 |
| I | Exame de base | | | Aplicação do repelente  duas vezes ao dia | | | Sem intervenção | | | | | | | | | | | | | | | | | | | Avaliação final | | |
| II |  |  |  |  |  |  | Cada segunda semana aplicação uma vez ao dia durante uma semana  (= 10 semanas de intervenção) | | | | | | | | | | | | | | | | | | |  |  |  |
| III |  |  |  |  |  |  | Cada quarta semana aplicação uma vez ao dia durante uma semana  (= 5 semanas de intervenção) | | | | | | | | | | | | | | | | | | |  |  |  |

Detalhamento da avaliação das medidas de desfecho durante o estudo:

| **Medidas de desfecho (semana)** | | | | | | | | | | | | | | | | | | | | | | | | | | | | |
| --- | --- | --- | --- | --- | --- | --- | --- | --- | --- | --- | --- | --- | --- | --- | --- | --- | --- | --- | --- | --- | --- | --- | --- | --- | --- | --- | --- | --- |
| coorte | 1 | 2 | 3 | 4 | 5 | 6 | 7 | 8 | 9 | 10 | 11 | 12 | 13 | 14 | 15 | 16 | 17 | 18 | 19 | 20 | 21 | 22 | 23 | 24 | 25 | 26 | 27 | 28 |
| I | 3x presença de lesões (sim/não)^a^  3x número total de lesões^b^  3x lesões estágio II e III  1x escore de severidade | | | 3x presença de lesões (sim/não)^a^  3x número total de lesões^b^  3x lesões estágio II e III  1x escore de severidade | | | Presença de lesões (sim/não)  Número total de lesões (uma vez por semana)  Lesões em estágio II e III (uma vez por semana)  Escore de severidade (uma vez ao mês) | | | | | | | | | | | | | | | | | | | | 2x presença de lesões (sim/não)^a^  2x número total de lesões^b^  2x lesões estágio II e III  1x escore de severidade | |
| II |
| III |
| Seis grupos de ratos Wistar sentinela: taxa de ataque a cada três dias | | | | | | | | | | | | | | | | | | | | | | | | | | | | |

^a^ →prevalência longitudinal

^b^ →intensidade de infestação

^c^ →proporção de lesões vitais

### Orçamento/financiamento

O estudo será financiado pela Fundação de Educação e Saúde Mandacaru, Fortaleza. Além disso, foi solicitado apoio através de edital universal do CNPq (Edital CNPq 19/2004). Nenhum dos pesquisadores será remunerado.

| Ingredientes para o repelente natural e manipulação |  | R$ 2000 |
| --- | --- | --- |
| Reuniões com a comunidade | 2 x R$ 150 | R$ 300 |
| Remuneração de três assistentes comunitários | R$ 240/mês (3 x 6 meses) | R$ 4320 |
| Fotodocumentação |  | R$ 500 |
| Material de escritório |  | R$ 500 |
| Cestas básicas | 100 x R$ 25 | R$ 2500 |
| Material e custos diversos |  | R$ 500 |
| Total |  | R$ 10620 |

**Grupo de pesquisa**

O estudo será realizado pelo grupo de pesquisa *Epidemiologia de doenças infecciosas e parasitárias no nordeste brasileiro*, linha de pesquisa *Epidemiologia, biologia, patologia, terapia e controle da tungíase* (cadastrado no CNPq com Prof. Jorg Heukelbach como líder) em cooperação com a Fundação de Educação e Saúde Mandacaru.

**Referências bibliográficas**

(1) Hoeppli R. Early references to the occurrence of *Tunga penetrans* in Tropical Africa. Acta Trop 1963;20(2):143-52.

(2) Gordon RM. The jigger flea. Lancet 1941;2:47-9.

(3) Guerra F. Aleixo de Abreu [1568-1630], author of the earliest book on tropical medicine describing amoebiasis, malaria, typhoid fever, scurvy, yellow fever, dracontiasis, trichuriasis and tungiasis in 1623. J Trop Med Hyg 1968 March;71(3):55-69.

(4) Hesse P. Die Ausbreitung des Sandflohs in Afrika. Geogr Z (Hettner) 1899;522-30.

(5) Henning G. Zur Geschichte des Sandflohs (*Sarcopsylla penetrans* L.) in Afrika. Naturwissenschaftliche Wochenschrift 1904;20:310-2.

(6) Hicks EP. The early stages of the jigger, *Tunga penetrans*. Ann Trop Med Parasitol 1930;24:575-86.

(7) Jeffreys MDW. *Pulex penetrans*: the jigger's arrival and spread in Africa. South African Journal of Science 1952;48:249-55.

(8) Heukelbach J, de Oliveira FA, Hesse G, Feldmeier H. Tungiasis: a neglected health problem of poor communities. Trop Med Int Health 2001 April;6(4):267-72.

(9) Matias RS. Combate ao bicho-de-pé começa no Sul. Ciência Hoje 1987;7(37):10-1.

(10) Wilcke T, Heukelbach J, Cesar Saboia MR, Regina SK-P, Feldmeier H. High prevalence of tungiasis in a poor neighbourhood in Fortaleza, Northeast Brazil. Acta Trop 2002 September;83(3):255-8.

(11) Carvalho RW, Almeida AB, Barbosa-Silva SC, Amorim M, Ribeiro PC, Serra-Freire NM. The patterns of tungiasis in Araruama township, state of Rio de Janeiro, Brazil. Mem Inst Oswaldo Cruz 2003 January;98(1):31-6.

(12) Muehlen M, Heukelbach J, Wilcke T, Winter B, Mehlhorn H, Feldmeier H. Investigations on the biology, epidemiology, pathology and control of *Tunga penetrans* in Brazil II. Prevalence, parasite load and topographic distribution of lesions in the population of a traditional fishing village. Parasitol Res 2003 May 27;90:449-55.

(13) Heukelbach J, Costa ALM, Wilcke T, Mencke N, Feldmeier H. The animal reservoir of *Tunga penetrans* in poor communities in Northeast Brazil. Med Vet Entomol 2004;in press.

(14) Eisele M, Heukelbach J, van Marck E, Mehlhorn H, Meckes O, Franck S et al. Investigations on the biology, epidemiology, pathology and control of Tunga penetrans in Brazil: I. Natural history of tungiasis in man. Parasitol Res 2003 June;90(2):87-99.

(15) Feldmeier H, Heukelbach J, Eisele M, Sousa AQ, Barbosa LM, Carvalho CB. Bacterial superinfection in human tungiasis. Trop Med Int Health 2002 July;7(7):559-64.

(16) Obengui. La tungose et le tétanos au C.H.U. de Brazzaville. Dakar Med 1989;34(1-4):44-8.

(17) Soria MF, Capri JJ. Tetanos y "piques". La Prensa Medica Argentina 1953;40(1):4-11.

(18) Tonge BL. Tetanus from chigger flea sores. J Trop Pediatr 1989;35:94.

(19) Litvoc J, Leite RM, Katz G. Aspectos epidemiológicos do tétano no estado de São Paulo (Brasil). Revista do Instituto de Medicina Tropical de São Paulo 1991;33(6):477-84.

(20) Bezerra SM. Tungiasis--an unusual case of severe infestation. Int J Dermatol 1994 October;33(10):725.

(21) Cardoso AEC. Tunguíase. An Bras Dermatol 1990;65(5a):29S-33S.

(22) Heukelbach J, Mencke N, Feldmeier H. Cutaneous larva migrans and tungiasis: the challenge to control zoonotic ectoparasitoses associated with poverty. Trop Med Int Health 2002 November;7(11):907-10.

(23) Ade-Serrano MA, Olomolehin OG, Adewunmi A. Treatment of human tungiasis with niridazole (Ambilhar): a double-blind placebo-controlled trial. Ann Trop Med Parasitol 1982 February;76(1):89-92.

(24) Cardoso A. Generalized tungiasis treated with thiabendazole. Arch Dermatol 1981;117:127.

(25) Valença ZO, Cardoso AEC, Cardoso AS. Tunguiase generalizada: relato de dois casos tratados com thiabendazol. Dermatologia Ibero-Latino-Americana 1972;3:375-8.

(26) Saraceno EF, Bazarra MLG, Calviello RC, Quaranta MA, Mirarchi P, Sánchez GF. Tungiasis: tratamiento de un caso con ivermectina. Arch Argent Dermatol 1999;49:91-5.

(27) Heukelbach J, Eisele M, Jackson A, Feldmeier H. Topical treatment of tungiasis: a randomized, controlled trial. Ann Trop Med Parasitol 2003 October;97(7):743-9.

(28) Heukelbach J, Franck S, Feldmeier H. Therapy of tungiasis: a double-blinded randomized controlled trial with oral ivermectin. Mem Inst Oswaldo Cruz 2004;99(8):873-6.

(29) Schwalfenberg S, Witt LH, Kehr JD, Feldmeier H, Heukelbach J. Prevention of tungiasis using a biological repellent: a small case series. Ann Trop Med Parasitol 2004 January;98(1):89-94.

(30) Heukelbach J, Wilcke T, Harms G, Feldmeier H. Seasonal variation of tungiasis in an endemic community. Am J Trop Med Hyg 2004;in press.

**TERMO DE CONSENTIMENTO LIVRE E ESCLARECIDO**

**PARA PARTICIPAÇÃO NA PESQUISA**

**“Aplicação intermitente de um repelente natural para reduzir a intensidade de infestação com *Tunga penetrans* e severidade de tungíase (*bicho de pé*) em uma área endêmica”**

Caro Sr./ Sra.

Estamos realizando uma pesquisa com a finalidade de saber se um repelente natural - produto à base de coco, aloe vera (babosa), jojoba, pantenol (derivado de vitamina B) e vitamina E – impede a penetração na pele de novas pulgas do bicho de pé. Essa pesquisa será importante para o controle dessa doença em comunidades severamente afetadas como a que o senhor/ a senhora reside.

Serão necessárias aplicações nos pés do repelente uma vez ao dia. O estudo tem uma duração total de 28 semanas. A cada aplicação serão realizadas perguntas sobre alguma reação que possa acontecer com o uso do repelente (coceira, ardor etc.) e contadas o número de novas pulgas que possam ter penetrado na pele. Eventualmente serão realizadas fotografias dos pés antes e após o uso do repelente.

É necessário esclarecer que:

1. A sua participação na pesquisa deverá ser de livre e espontânea vontade.
2. Ao participar da pesquisa, o risco para a saúde é mínimo (as reações na pele com o uso do repelente são raras e ele já é comercializado como repelente contra mosquitos e carrapatos).
3. A sua identificação será mantida em segredo.
4. O senhor/ a senhora poderá desistir de participar, a qualquer momento, sem qualquer prejuízo de sua assistência.
5. Será permitido o livre acesso a informações pertinentes à pesquisa.
6. Somente após devidamente esclarecido e ter entendido o que foi explicado, deverá o senhor/ a senhora assinar esse documento, caracterizando a sua autorização para participar da pesquisa.

Em caso de dúvida, poderá comunicar-se com o pesquisador responsável:

Prof. Jorge Heukelbach

Departamento de Saúde Comunitária da Faculdade de Medicina (UFC)

Rua Prof. Costa Mendes 1608, 5° andar

CEP: 60.430-140

Tel: 3288 8044 ou 3288 8045

O Comitê de Ética em Pesquisa encontra-se disponível para reclamações pertinentes à pesquisa pelo telefone 3288 8346.

Fortaleza, ______de ___________________de 2005.

Nome completo do participante: _____________________________________________________________

___________________________________________

Assinatura do sujeito da pesquisa

___________________________________________

Assinatura do responsável legal (se menor de idade)

___________________________________________

Jorg Heukelbach (pesquisador)
