# Supplementary material for: Control of Tungiasis through Intermittent Application of a Plant-Based Repellent: An Intervention Study in a Resource-Poor Community in Brazil
Source: PLoS Negl Trop Dis. 2010 Nov 9;4(11):e879. doi: 10.1371/journal.pntd.0000879 (PMC2976681; doi:10.1371/journal.pntd.0000879)
Supplement: Text S1 — Compounds of Zanzarin. (0.02 MB DOC) [file pntd.0000879.s003.doc]

**Technical Annex:**

**Compounds of Zanzarin :**

**Refined coconut oil** (*Cocos nucifera*)

**Jojoba ester** (extracted from semen of *Simmondsia chinensis*)

**Aloe** (extracted from *Aloe barbadensis* leafes**)**

**Tocopheryl acetate**

**Capric acid**

**Panthenol, Methylparaben, Propylparaben** (Preservative)
